# Monocyte Trafficking and Polarization Contribute to Sex Differences in Meta-Inflammation

**DOI:** 10.3389/fendo.2022.826320

**Published:** 2022-03-28

**Authors:** Mita Varghese, Jeremy Clemente, Arianna Lerner, Simin Abrishami, Mohammed Islam, Perla Subbaiah, Kanakadurga Singer

**Affiliations:** ^1^ Department of Pediatrics, Michigan Medicine, University of Michigan, Ann Arbor, MI, United States; ^2^ Department of Statistics and Mathematics, Oakland University, Rochester, MI, United States

**Keywords:** obesity, monocyte, macrophage, sex differences, metabolism

## Abstract

Obesity is associated with systemic inflammation and immune cell recruitment to metabolic tissues. Sex differences have been observed where male mice challenged with high fat diet (HFD) exhibit greater adipose tissue inflammation than females demonstrating a role for sex hormones in differential inflammatory responses. Circulating monocytes that respond to dietary lipids and chemokines and produce cytokines are the primary source of recruited adipose tissue macrophages (ATMs). In this study, we investigated sexual dimorphism in biological pathways in HFD-fed ATMs from male and female mice by RNA-seq. We also conducted chemotaxis assays to investigate sex differences in the migration of monocytes isolated from bone marrow from male and female mice toward a dietary saturated lipid — palmitate (PA), and a chemokine — monocyte chemoattractant protein 1 (MCP1), factors known to stimulate myeloid cells in obesity. ATM RNA-Seq demonstrated sex differences of both metabolic and inflammatory activation, including pathways for chemokine signaling and leukocyte trans-endothelial migration. *In vivo* monocyte transfer studies demonstrated that male monocytes traffic to female adipose tissue to generate ATMs more readily. In chemotaxis assays, lean male monocytes migrated in greater numbers than females toward PA and MCP1. With short-term HFD, male and female monocytes migrated similarly, but in chronic HFD, male monocytes showed greater migration than females upon PA and MCP1 stimulation. Studies with monocytes from toll-like receptor 4 knockout mice (*Tlr4^-/-^*) demonstrated that both males and females showed decreased migration than WT in response to PA and MCP1 implying a role for TLR4 in monocyte influx in response to meta-inflammation. Overall, these data demonstrate the role of sexual dimorphism in monocyte recruitment and response to metabolic stimuli that may influence meta-inflammation in obesity.

## Introduction

Immune systems of men and women have several differences in innate and adaptive immunity and hematopoietic responses (1, 2). Men have a higher susceptibility to a variety of pathogens leading to an increased frequency of infectious diseases (3), while women have a higher rate of autoimmune diseases (4). Females, compared to males, show a stronger humoral and cell-mediated immunity (5, 6), as demonstrated by higher levels of immunoglobulins (7) as well as stronger antibody responses to viral vaccines (5). The production of cytokines and chemokines by innate immune cells also differs between the sexes. In addition to the role of sex and sex hormones influencing leukocyte biology, sexual dimorphism is also observed in responses to a variety of stressors including dietary fats and lipid metabolism. This role of sex effects on immune responses has been proposed as a mechanism that differentiates the risk of cardiovascular and metabolic disease in men versus women (8). In women, estrogen has been proposed to be protective against atherosclerotic cardiovascular disease (CVD). This view is supported by the increase in CVD risk in women seen after menopause, which involves a natural decline in ovarian hormone production (9). The role of sex in modifying inflammatory responses, specifically monocyte/macrophage responses, may explain the dimorphism observed in this disease risk.

Sex is a major factor determining adipose tissue distribution and accumulation and the subsequent adverse effects of obesity-related diseases including type 2 diabetes and CVD (10). Our understanding of metabolic inflammation stems from studies of mice fed with high fat diet (HFD) enriched with saturated fatty acids (SFAs). HFD drives increased weight, adipose expansion, and macrophage polarization. In the lean adipose tissue, resident adipose tissue macrophages (ATMs) are the predominant macrophage subset that function to maintain homeostasis and are distinguished by a CD64^+^/CD11c^−^ phenotype (11, 12). During HFD exposure, there is an early increase in macrophages with initial proliferation of resident ATMs (13–15) in the obese adipose tissue. With the expansion of the visceral white adipose tissue (WAT) in obesity, there is a profound accumulation of the pro-inflammatory CD11c^+^ ATM population, particularly in males, while females show dampened inflammation (16, 17). Circulating Ly6c^hi/^CCR2^+^ blood monocytes recruited to the adipose tissue are largely considered a predominant source for ATM accumulation and develop into CD11c^+^ ATMs (18, 19). These ATMs are a prominent source of inflammatory cytokines, such as interleukin (IL)1β, IL6, and tumor necrosis factor α (TNFα) (20–22) and chemokines such as MCP1/CCL2 (17, 23) that are important contributors to insulin resistance and overall metabolic syndrome in obesity (7, 24).

Understanding the sexual dimorphism of monocyte to macrophage transition may further explain how to best target metabolic inflammation in males and females. Studies have demonstrated that leukocytes in women have lower expression of toll-like receptor 4 (TLR4) and lower cytokine production, clinically demonstrated by reduced sepsis rates among women (25). Peripheral blood mononuclear cells (PBMCs) from men produce more pro-inflammatory TNFα and less protective IL10 than PBMCs from women following LPS stimulation (20). Peritoneal macrophages from male mice express higher levels of TLR4 and produce more CXC-chemokine ligand 10 (CXCL10) following LPS stimulation than macrophages from females (25), demonstrating the importance of several factors in regulating chemotaxis and recruitment in inflammatory responses. Beyond LPS models, sexual dimorphism has been demonstrated in viral assays with PBMCs from men resulting in higher TLR9 activation and increased IL-10 production, which positively correlated with androgen concentrations in males (26). The myeloid pools themselves are also larger and may traffic more readily in males compared to females (21). Sex differences in macrophage polarization in visceral/gonadal WAT (GWAT) are related to diet-induced differences in hematopoiesis, lipid metabolism, and polarization (22). Evaluation of monocytes and hematopoietic progenitors in diet-induced obesity models shows that females do not have an expansion of the myeloid progenitors with HFD. *Ex vivo* studies of bone marrow (BM) from female mice stimulated with LPS or palmitic acid (PA) also showed lower pro-inflammatory cytokine expression levels compared to males (17). In competitive bone marrow transplant (BMT) experiments, where recipients received both male and female bone marrow in a 1:1 ratio, male BM cells showed enhanced production of CD11c^+^ ATMs in response to HFD. Overall, female mice are protected from HFD-induced reprogramming of HSCs, ATM accumulation, and insulin resistance, similar to premenopausal women with obesity (23, 24, 27). However, there is a gap in understanding the phenotypic differences in monocyte trafficking, activation, and polarization in males and females.

Here, we identified sex differences in macrophage chemokine receptors and chemokine production through RNA sequencing of ATMs from male and female mice. To further investigate sex differences in diet-induced monocyte responses, we compared the migration efficiency of male and female monocytes. In this study, we also demonstrate sex differences in monocyte migration and in their response to SFA and chemokine production following HFD. Male monocytes showed migration in greater numbers than females toward PA and MCP1 in lean conditions. Assessment of inflammatory chemokines showed higher levels of MCP1 in males than females with HFD. Overall, these data demonstrate that male monocytes are more inflammatory in nature than female monocytes. Compared to female monocytes, male monocytes respond more robustly to metabolic stimuli with increased monocyte recruitment and are more likely to mature into inflammatory macrophages than females. A better understanding of sex differences with respect to monocyte responses may contribute to sex-based intervention studies for prevention and treatment of obesity and related diseases.

## Materials and Methods

### Animals

C57Bl/6J (WT) and male and female mice were purchased from Jackson Laboratories (000664) at 5 weeks of age. Animals were housed in a pathogen-free facility and at 6 weeks were either maintained on normal chow diet (ND) (5L0D, 13.5% fat, Lab Diets) or started a HFD chow (D12492: 60% fat, Research Diets) for the specified length of time. *Tlr4* ***^-/-^*** (B6.B10ScN/JthJ; 007227) mice derived from Jackson Laboratories were used for trafficking studies with TLR4 knockout. Animal protocols were in compliance with the Institute of Laboratory Animal Research Guide for the Care and Use of Laboratory Animals and approved by the University Committee on Use and Care of Animals at the University of Michigan.

### Bone Marrow Monocyte Isolation and Migration/Chemotaxis Assay

Monocytes were prepared from bone marrow (BM) of C57Bl/6J (WT) and *Tlr4* ***^-/-^*** male and female mice. Mice were sacrificed and hip, femur, and tibia were obtained. To isolate the BM, the bones were flushed with RPMI-1640 (Gibco 21870-076) plus Pen strep antibiotic (Gibco 15270-063). Next, monocytes were isolated from BM cells using Stem Cell EasySep mouse monocyte isolation kits (Cat # 19861) as per the manufacturer’s protocol.

Migration/chemotaxis assay was performed with Corning 24-well plates containing Boyden filter chambers (Cat. # 3421, pore size 5 μm). Bottom chamber media consisted of DMEM (Gibco #11965-092) supplemented with 10% fetal bovine serum (FBS; Invitrogen #10082-147) and 1% pen strep (P/S). Upper chamber media contained DMEM + 1% FBS + 1% P/S. A total of 500,000 cells were added to 200 μl of upper chamber media per well. In the bottom chamber media, for experiments, BSA was used as control or PA (10 μM/ml) plus MCP1 (100 ng/ml) was added. This dosage of PA was determined after a titration curve found to be the best dosing for *in vitro* studies (17). Plates were incubated for 6 h, and thereafter, nonmigrated cells were removed gently from the top of the filter with a cotton swab. The filter was washed twice by pipetting 250 μl RPMI (without FBS) to ensure the removal of any nonmigrated cells. Thereafter, cells that had migrated to the bottom of the filter as well as cells that had migrated to the bottom of the plate were trypsinized for 15 min. The cells were then washed and collected with PBS and centrifuged for 5 min at 500 g. The pelleted cells were resuspended in 100 μl of PBS and counted using a hemocytometer.

### Fatty Acid and Chemokine Preparations

Palmitic acid (Sigma, #P5585) (PA) was prepared in isopropanol at a stock concentration of 50 mM and then complexed with 10% BSA (endotoxin free, fatty acid free; Sigma #A8806) in isopropanol to make up 5 mM. Fatty acid–free BSA was further used as the control in experiments to rule out endotoxin contamination of BSA as the source of palmitic acid’s effects as reported earlier in some cases (28, 29). MCP1 (R&D 479 JE) was used at a concentration of 100 ng/ml of media. Stock solution of MCP1 was prepared in PBS at a concentration of 50 μg/ml.

### Monocyte Transfer Studies

BM was flushed from the femurs and tibia of CD45.1 (002014 Jackson Laboratories) or CD45 1.2 mice bred from CD45.1 (crossed with wild-type C57Bl6/J), and monocytes were isolated with a Magnetic Miltenyi Monocyte Isolation Kit (130-100-629). A total of 500,000 cells were isolated and injected into male or female CD45.2 mice placed on HFD for 2 weeks before isolation of tissues. CD45.1 PercP5.5 (A20) antibodies were added to the below flow cytometry stains to identify donor monocytes.

### Adipose Tissue Stromal Vascular Fraction (SVF) Isolation and Flow Cytometry

Adipose tissue fractionation and flow cytometry analysis were performed as described previously (17). Briefly, whole adipose tissue was minced and digested with type II collagenase (Sigma 1 mg/ml in RPMI media) for 15–30 min at 37°C on a rocker. Filtrated samples were spun at 500g for 10 min and RBC lysis conducted (biosciences 00-4333-57). SVF cells were stained with antimouse CD45 eFluor450 or APC (30-F11 monoclonal, *Invitrogen*), CD64 PE (X54-5/7.1 monoclonal, BD Pharmingen), and CD11c APC or eFluor 780 (N418 monoclonal, Invitrogen), and gating was performed for macrophage populations and by CD45 gates to determine ATMs (30). Flow cytometry was conducted on BD Canto or Aria III machines and analyzed on the FlowJo software. For sorting, 10 min of DAPI staining was performed for live/dead staining for ATM isolation. After washing, collection tubes with 100 μl of FACS buffer were used to collect cells. Following collection, cells were spun down and RNA prepared from cell pellets.

### ELISA

ELISA was performed for cytokine determination of serum MCP1 and CCL5 levels from mice on control chow or HFD for 2, 6, and 16 weeks. Testing was performed by the Cancer Center Immunology core at the University of Michigan.

### Quantitative Real-Time PCR

RNA was extracted from adipose tissue using Trizol LS (Life Technologies) or sorted cells/monocytes were prepared using a Qiagen RNA kit (#74106; Qiagen). cDNA was generated using a High-Capacity cDNA Reverse Transcription Kit (Applied Biosystems). SYBR Green PCR Master Mix and the StepOnePlus System (Applied Biosystems) were used for real-time quantitative PCR. *Arbp* expression was used as an internal control for data normalization. Samples were assayed in duplicate, and relative expression was determined using the 2-ΔΔ CT method. All primers used are listed in **Supplementary Table 1**.

### RNA-Sequencing

**Figure 1:**
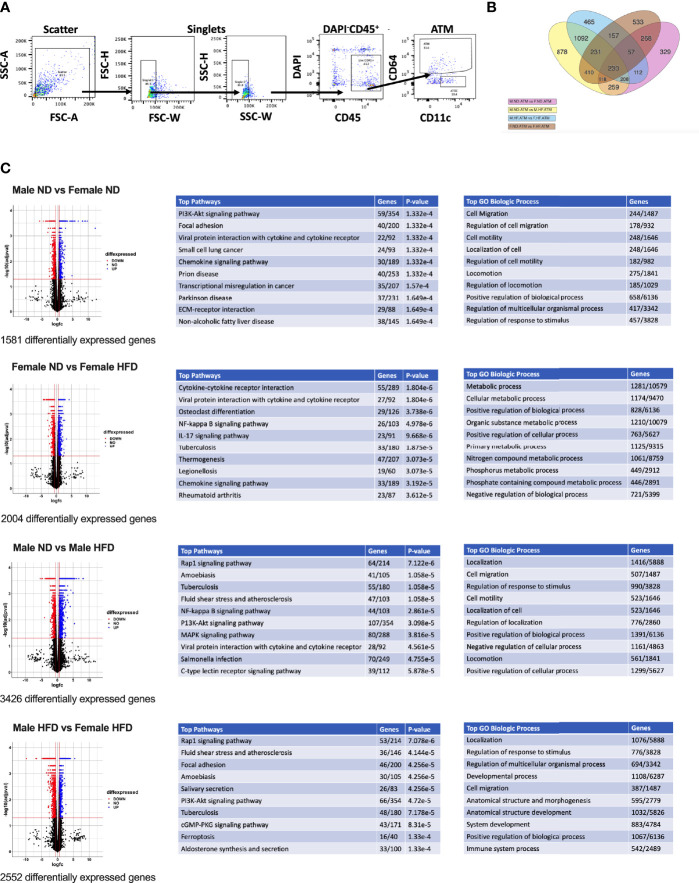
RNA signatures of male and female ATMs. RNA-seq was conducted from **(A)** FACS sorted male and female ATMs (PI^-^, CD45^+^, CD64^+^) from normal diet (ND) and high fat diet (HFD) for 16 weeks. **(B)** Venn diagram of the number of significantly different genes in ATM comparisons of male ND vs male HFD, female ND vs female HFD, male ND vs female ND, and male HFD vs female HFD. **(C)** Volcano plots of differential genes and top 10 pathways and GO Biologic Process. N = 4 per group.

The RNA-sequencing experiment consisted of 4 groups with 4 replicates per group; groups were defined by gender (M/F) and diet (ND/HFD). Following cell sorting, cells were pelleted, and RNA was prepared using a Qiagen RNA kit. Standard practice in the sequencing core is to perform quality control for concentration and RNA integrity number. After QC, library preparation and sequencing was also performed at the University of Michigan Advanced Genomics Core. Libraries were prepared with the Takara/Clonetech SMARTer standard kit, after ribodepletion with RiboGene, resulting in 125 base fragments. Sequencing was done on the Illumina Hi-Seq 4000 platform, with 50 single-end cycles. Fastq read files were uploaded for analysis. Data analysis was performed by the University of Michigan Bioinformatics Core. FastQC (version v0.11.3) was used for quality control before and after alignment, and all samples passed. The Tuxedo Suite was used for alignment, differential expression analysis, and post-analysis diagnostics (31–33). TopHat (version 2.0.13) and Bowtie2 (version 2.2.1) were used for alignment based on the UCSC mm10 reference. Cuttlinks/Cutt Diff (version 2.1.1) was used for expression quantitation, normalization, and differential expression analysis. To identify a given gene as differentially expressed (DE), the cutoffs were as follows: absolute fold change of at least 1.5, and FDR corrected p-value less than or equal to 0.05. Pathway and gene ontology analysis (**Figure 1**) was performed in Advaita iPathways. Data are publicly available on NCBI GEO accession GSE181841.

**Figure 2:**
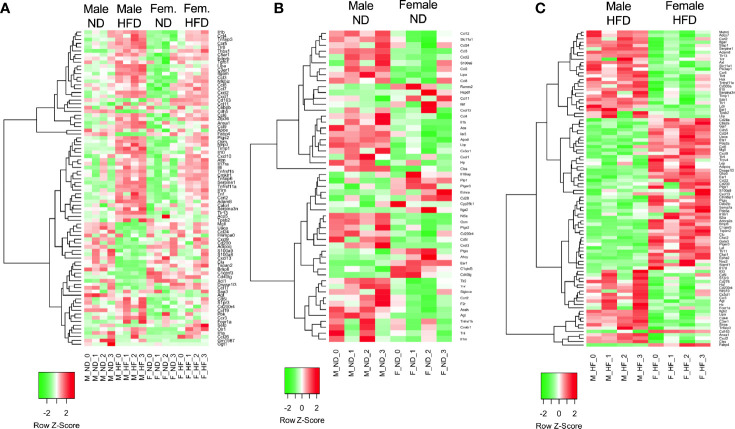
RNA signatures differ in the GO term of inflammatory responses. RNA-seq from male and female ND and HFD ATMs that had significant differences between males and females [log2(fold change)] in the GO term inflammatory responses were plotted in heatmaps. **(A)** All groups, **(B)** ND animals, and **(C)** HFD animals. N = 4 per group.

Volcano plots were generated in the R program (version 4.1.2) using ggplot2 (34). Log fold change and FDR-corrected p-values were imported into R using readxl (35). All genes with an FDR-corrected p-value = 1 were excluded. Heatmaps were prepared in unsupervised and supervised manner. Both used all male and female samples on either ND or HFD, starting from the matrix of RPKM normalized values. Both heatmaps were generated with the heatmap.2 function in gplots R package, using the default distance (Euclidean), and Ward.D2 agglomeration. The genes were z-score-scaled for heatmap display. For the unsupervised heatmap (**Figure 2**), the subset of genes to plot was selected to have high variance and high base expression, specifically genes having a maximum expression of at least one RPKM, and at least one standard deviation, over all samples. These filters resulted in 84 DE genes, selected independently of differential expression results. For the supervised heatmap, the subset of genes to plot was selected from the DE genes from the GO term related to Inflammatory Response GO 0006954 and all of its child terms (84 GO terms and 651 unique gene symbols related to immune response, based on the January 2019 Gene Ontology release).

### Statistical Analysis

The data from each experiment were analyzed using t-tests and, when appropriate, 2-way ANOVA accounting for diet/treatment and sex, with *post hoc* analysis using Tukey’s multiple comparisons test as specified in each figure in the Prism 9 software. Data are shown as average +/- SEM in the figures.

## Results

### Male and Female ATMs Differ in RNA Signatures of Metabolic and Inflammatory Gene Pathways

C57Bl/6J mice were placed on 60% HFD at 6 weeks of age for a duration of 16 weeks. Control animals were maintained on ND for comparison. After this length of time on diet, both male and female animals fed with HFD had increased weight gain and visceral gonadal white adipose tissue weight (GWAT) expansion (**Supplementary Figure 1A**). Among mice on HFD, only males had significant increases in fed glucose levels (**Supplementary Figure 1A**). To determine sex differences in macrophage function, ATMs (PI^-^, CD45^+^, CD64^+^) were sorted from GWAT of both male and female lean and obese mice and examined by RNA-seq (**Figure 1A**). Primary differential gene analysis was conducted on comparisons based on sex (male ND vs female ND and male HFD vs female HFD) and diet (female ND vs female HFD and male ND vs male HFD) (**Figure 1B**). **Supplementary Figures 1B** and **2** show comparisons of top 20 genes that were upregulated or downregulated among the different groups. Top differential pathways and biological processes observed in male ND vs female ND were related to cell motility, migration, and adhesion as well as activation of the PI3K-Akt pathway, which has been associated with monocyte/macrophage polarization (36, 37) (**Figure 1C**). In addition to direct comparisons between groups (**Figures 1B, C**), we also assessed top biological processes that drive male macrophages by sorting M HFD vs FHFD followed by M ND vs M HFD. Several biological pathways were found to be differentially regulated between male and female GWAT ATMs (**Table 1**). Interestingly, sex differences were specifically observed in both ATM fat storage (regulation of lipolysis, carbohydrate digestion) and inflammation (hematopoietic cell lineage, chemokine, cytokine–cytokine interactions) pathways. Given our primary interest in inflammatory responses, heatmaps were generated to evaluate the specific genes under the pathway of inflammatory response (**Figures 2A–C**). ATMs from obese males upregulated gene pathways for inflammation, hematopoiesis, fatty acid metabolism, and insulin resistance (*Il1b, Tlr8*, and *Lipa* to name a few listed in **Figure 2A**). Sexual dimorphism was observed in the cluster for inflammatory responses in ND animals (**Figure 2B**) as well as in HFD, where genes were downregulated in the female HFD ATMs (*Ccr12, Tnf, Lbp*, and *Ldlr)* (**Figure 2C**). Heatmaps shown in **Figures 2A–C** show genes significantly changed with a minimum expression [log2(FPKM+1)] of 1 and a minimum log2(fold change) of 1 between sexes in each diet group under the GO cluster of inflammatory responses. These results emphasize that there are intrinsic sex-specific differences in myeloid cell-activated states that may contribute to both metabolic and inflammation differences in adipose tissue.

**Table 1:** P-value for top biological pathways after FDR sorting for M HFD vs F HFD and M ND vs M HFD.

Pathway name	M ND vs M HFD	M HFD vs F HFD
**Lysosome**	3.32E-20	3.20E-16
**Phagosome**	7.66E-10	3.78E-9
**Metabolic pathways**	2.82E-8	3.40E-8
**Hematopoietic cell lineage**	1.83E-4	1.41E-7
**Cell adhesion molecules**	5.09E-6	5.55E-7
**Rap1 signaling pathway**	8.68E-7	5.75E-7
**Pathways in cancer**	1.90E-6	1.23E-6
**Fluid shear stress and atherosclerosis**	4.87E-6	6.25E-6
**Glutathione metabolism**	2.75E-4	7.21E-6
**PI3K-Akt signaling pathway**	1.10E-5	9.58E-6
**Focal adhesion**	4.20E-5	9.58E-6
**Salivary secretion**	5.10E-4	1.72E-5
**Axon guidance**	5.57E-5	2.00E-5
**Tuberculosis**	5.09E-6	3.91E-5
**Transcriptional mis-regulation in cancer**	5.19E-5	4.37E-5
**Amoebiasis**	3.20E-6	4.58E-5
**cGMP-PKG signaling pathway**	3.30E-3	4.87E-5
**Rheumatoid arthritis**	1.49E-4	7.42E-5
**Aldosterone synthesis and secretion**	1.42E-4	7.94E-5
**Leukocyte trans-endothelial migration**	1.54E-4	7.94E-5
**Cytokine-cytokine interaction**	4.55E-4	1.02E-4
**Ferroptosis**	1.59E-3	1.09E-4
**Gastric acid secretion**	5.49E-4	1.46E-4
**Vascular smooth muscle contraction**	2.30E-3	1.81E-4
**NF-kappa B signaling pathway**	1.10E-5	1.82E-4

### Sex Differences in Chemokine Signatures and Receptors

**Figure 3:**
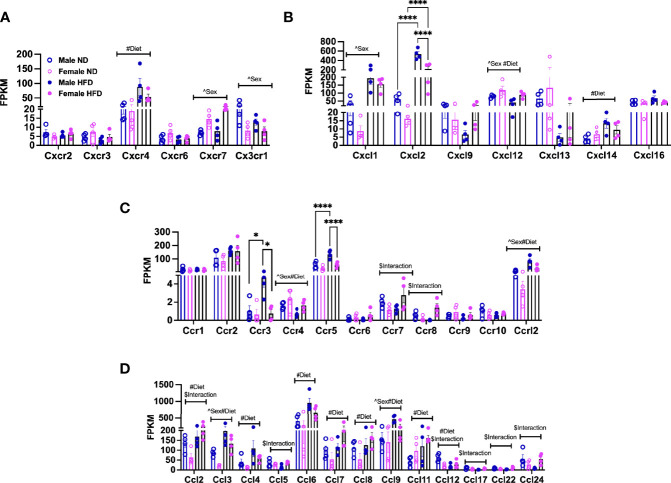
Sex differences in gene expression of ATM receptors and ligands. FPKM counts from single-cell RNA-seq data showing gene expression of **(A)** chemokine receptors, **(B)** chemokine ligands, **(C)** chemokine (C-C motif) receptors, and **(D)** chemokine (C-C) motif ligand. N = 4 per group. Data were analyzed by 2-way ANOVA followed by Tukey’s multiple comparisons test. Data shown as average +/- SEM. *p < 0.05, and ****p < 0.001. Figures are marked for significance of main effects (^sex or #diet) and $interaction.

Chemokines and their receptors are noted to have significant redundancy (38), but an understanding of differences by sex with diet is helpful for understanding the dimorphism (39). Among the top differentially expressed genes in pathways associated with cell adhesion, migration, and interaction, we looked at markers for pro-inflammatory states of ATMs in heatmaps. We identified that several chemokines and chemokine receptors were listed among those. We plotted the fragments per kilobase millions (FPKMs, normalized reads) for CXC/CX3C receptors (**Figure 3A**, significant effects of diet, sex, and interaction—**Supplementary Table 2**) and ligands (**Figure 3B**, significant effect of diet, sex, and interaction—**Supplementary Table 2**) and CC receptors (**Figure 3C**, significant effect of diet, sex, and interaction—**Supplementary Table 2**) and CC ligands (**Figure 3D**, significant effect of diet, sex, and interaction—**Supplementary Table 2**). *Cxcr4, Cxcl14, Ccl4, Ccl6, Ccl7, Ccl8, and Ccl11* gene expressions were all observed to be increased with HFD in both sexes (**Figures 3A–D**). Others were seen to only be altered by sex; some were increased in females (*Cxcr7*), while others were increased in males (*Cx3cr1*) (**Figure 3A**). Some had main effect differences of both sex and diet (*Cxcl12, Ccr4, Ccr12, Ccl3, Ccl9*), while others had significant interactions (*Ccr7, Ccr8, Ccl5, Ccl17, Ccl24*) (**Figures 3A–D** and **Supplementary Table 2**). Some genes were of particular interest given changes by diet with a significant interaction (*Ccl2* and *Ccl12*) (**Figure 3D**). Genes significantly different by sex, diet, and interaction included *Cxcl2, Ccr3*, and *Ccr5*, which were all higher in male HFD compared to female HFD (**Figures 3B, C**).

Overall, these gene expression patterns demonstrate that many chemokines and receptors are altered by HFD treatment. Additionally, the results suggest that male and female monocytes have some similarities in chemokine responses but also have distinct expression differences that may contribute to inflammatory responses and tissue inflammation.

### Male Monocytes Remain Primed to Produce CD11c^+^ Macrophages in Response to HFD, Even in Female Animals

Previous *in vitro* studies from our lab showed that male BM cells differentiate into activated macrophages to a greater extent compared to female BM in culture (17). Also, a prior study from our lab utilizing BMT (male BM and female BM cells in a 1:1 ratio injected into male or female recipients) demonstrated that male BM was primed to produce CD11c^+^ ATMs compared to female BM in either recipient (17). While it is known that the initial response to HFD during adipose expansion is through local macrophage proliferation (40), there is also monocyte recruitment into the expanding adipose tissue wherein injected monocytes can home to the tissue.

**Figure 4:**
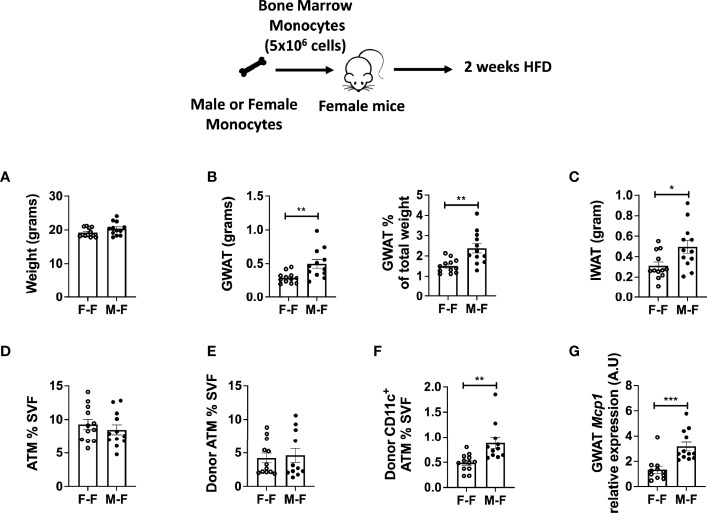
Monocyte transfer demonstrates retained inflammatory profile in male myeloid cells. Bone marrow monocytes from either female (F-F) or male (M-F) animals were next transferred into females that were then treated with 2 weeks of HFD. End-point assessments were made in **(A)** weight, **(B)** GWAT, % GWAT of total body weight, and **(C)** IWAT. Flow cytometry assessments of **(D)** ATM % of SVF, **(E)** donor ATM %, **(F)** donor-specific CD11c^+^ ATMs. **(G)** RT-PCR determined increased *Mcp1* in animals given male monocytes. N = 11–12 per group. Data were analyzed by two-sample t-test. Data shown as average +/- SEM. *p < 0.05, **p < 0.01 and ***p < 0.005.

To examine whether male monocytes have the ability to promote inflammatory CD11c^+^ ATMs in a female environment, we performed adoptive transfer experiments in which male or female monocytes were injected into female non-irradiated animals. These animals were then challenged to 2 weeks of HFD starting at 10 weeks of age. While animals were of equal weight (**Figure 4A**), recipients receiving male monocytes had larger GWAT (**Figure 4B**) and inguinal WAT (IWAT) pads (**Figure 4C**). Even though total ATMs were similar (**Figures 4D, E**), there were significantly more CD11c^+^ ATMs of male donor origin (tracked by CD45.1 donor marker) than those that received female monocytes (**Figure 4F**). Chemokine gene expression showed that *Mcp1* mRNA was elevated in GWAT of mice receiving male monocytes (**Figure 4G**), suggesting that BM-derived monocytes are partly responsible for the sex differences in ATM accumulation in response to HFD.

### Male Monocytes Have Increased Chemotaxis Compared to Female Monocytes in Response to Dietary Lipid and Chemokine Exposure

**Figure 5:**
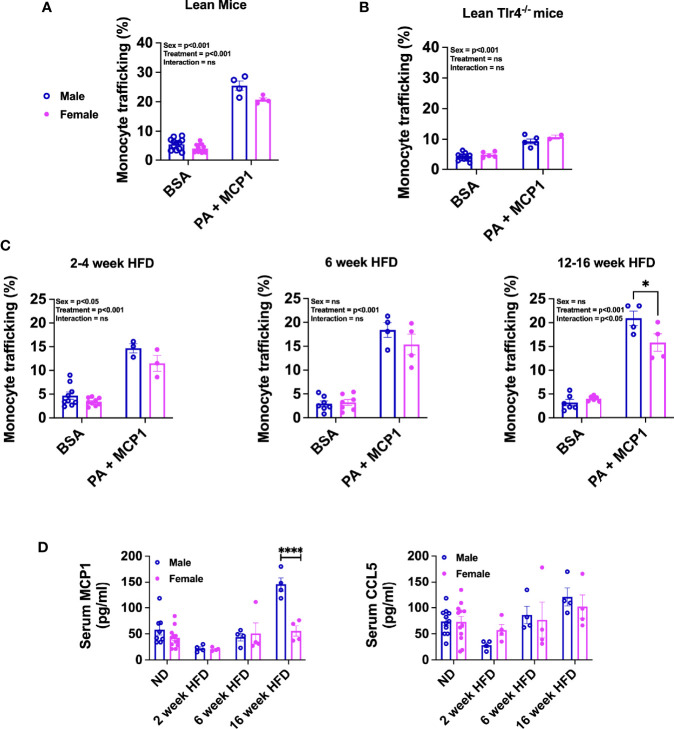
Sex differences in migration efficiency of monocytes with HFD. Monocyte trafficking (%) of BM monocytes from male and female **(A)** lean 22-week-old mice and **(B)** lean 22-week-old *Tlr4^-/-^* mice. Monocytes stimulated with a combination of PA and MCP1 in **(C)** 2- to 4-week HFD mice, 6-week HFD mice, and 12- to 16-week HFD mice. N=4-6 mice per condition. Data were analyzed by 2-way ANOVA followed by Tukey’s multiple comparisons test. **(D)** Serum ELISA from ND, 2-, 6-, and 16-week HFD male and female of **(D)** MCP1 and CCL5. These data were analyzed by two-sample t-test. N = 12 for ND; 4 for HFD. Data shown as average +/- SEM. *p < 0.05, and ****p < 0.001. ns= non-significant.

Studies with obese mice models have demonstrated that monocyte recruitment and macrophage accumulation in the adipose tissue are dependent on metabolic stimuli such as cytokines, chemokines, and dietary lipids and are sexually dimorphic (11, 16, 17). An *in vitro* monocyte trafficking system was used to assess sex differences in monocyte trafficking. BM monocytes were isolated from lean male and female 22-week-old C57Bl/6J mice and plated. After 6 h of incubation, monocytes that migrated to the bottom of the Boyden chamber were counted. Both male and female monocytes showed a similar response to BSA; however, with combination treatment of PA and MCP1, male monocytes trafficked at a higher frequency than females (**Figure 5A**, p=0.0561 for interaction and significant by treatment and sex—**Supplementary Table 3**). To assess the role of TLR4 in monocyte trafficking, BM monocytes from lean *Tlr4^-/-^* male and female mice were also tested. *Tlr4^-/-^* male and female BM monocytes overall trafficked at lower rates with no significant changes in trafficking efficiency at baseline or with the combination treatments of PA and MCP1 in either sex (**Figure 5B**, significant difference for sex but not by treatment or interaction—**Supplementary Table 3**) suggesting a role for TLR4 in monocyte recruitment into the adipose tissue in obese conditions.

Furthermore, we investigated the impact of short- and long-term HFD exposure on migration efficiency of monocytes. With short-term diet challenge of 2–4 weeks, monocyte trafficking was significantly increased with treatment and differed by sex (**Figure 5C**; left, no significant interaction—**Supplementary Table 3**). With longer-term HFD of 6 weeks, trafficking normalized among both sexes with similar increases with treatment (**Figure 5C**; middle, significant treatment effect with no significant interaction or sex effect—**Supplementary Figure 3**). When BM monocytes from long-term 12–16 week HFD-fed mice were evaluated, we observed a significant interaction with females having lower monocyte trafficking than males (**Figure 5C**; right and **Supplementary Figure 3**). To determine any significant changes in chemokine protein expression upon HFD exposure, MCP1/CCL2 and CCL5 were measured in the serum of mice along a time course of HFD. MCP1 and CCL5 are key chemokines that are upregulated in obesity and function to recruit monocytes to inflammatory sites (41, 42). Interestingly, in our HFD studies, we observed that MCP1 was significantly elevated in males but only with chronic HFD (**Figure 5D**). However, CCL5 levels did not reach a statistical significance in any of the groups.

**Figure 6:**
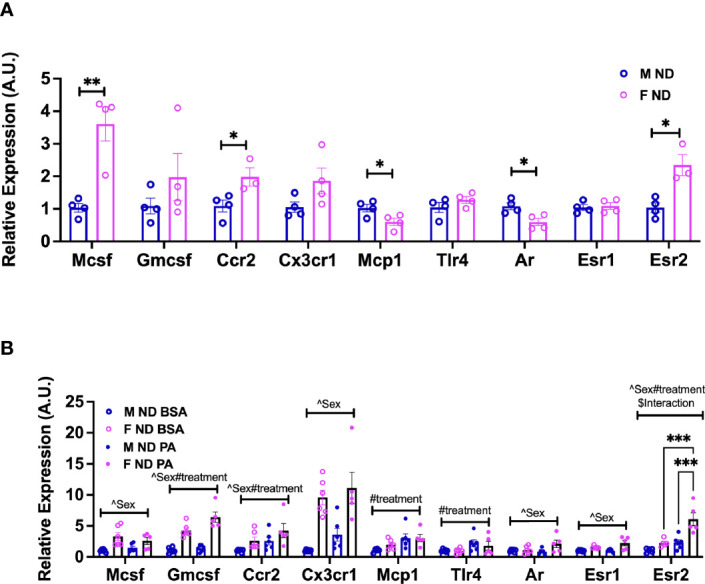
Sex differences in the gene expression of monocyte markers upon stimulation with a SFA and chemokine. BM male and female monocyte gene expression of *Mcsf, Gcsf, Ccr2, Cx3cr1, Mcp1, Tlr4, Ar, Esr1*, and *Esr2* from **(A)** 13-week-old mice. Data were analyzed by two-sample t-test. **(B)** 22-week-old mice. N = 4–6 per condition. Data were analyzed by 2-way ANOVA followed by Tukey’s multiple comparisons test. Data shown as average +/- SEM. *p<0.05, **p<0.01 and ***p<0.005. Figures are marked for significance of main effects (^sex or #treatment) and $interaction.

Nagashimada et al. showed that CX3CL1-CX3CR1 signaling deficiency exacerbates obesity-induced inflammation and insulin resistance in male mice (43), while Lesnik et al. (44) showed a role for CX3CR1 deficiency in decreased atherosclerosis. Myeloid-stimulating factors are important both for monocyte function and for continued generation of myeloid cells. MCSF is a potent mediator of monocyte and macrophage activation increasing their chemotactic and phagocytic functions (45). MCSF also stimulates the production of several cytokines including GMCSF, IL1, IL6, TNFα, and interferon (46). To evaluate any sex differences in receptor responses in monocytes compared to our earlier ATM RNA-seq data, we next performed gene expression analysis in isolated BM monocytes from males and females. At 13 weeks of age, we observed that isolated female monocytes showed a significantly higher expression of *Mcsf* and *Ccr2* (**Figure 6A**). On the other hand, *Mcp1/Ccl2* expression was higher in males than female monocytes (**Figure 6A**). We further assessed monocyte receptor gene expression in mice at 22 weeks of age along with PA stimulation for 6 h to assess sex differences in inflammatory markers. Interestingly, female monocytes showed higher expression of *Mcsf, Gmcsf*, and *Cx3cr1* than males at 22 weeks of age. There were significant sex effects in the gene expression of *Mcsf, Gmcsf, Ccr2*, and *Cx3cr1* (**Figure 6B** and **Supplementary Table 4**). Upon PA stimulation, treatment effect differences were seen in *Gmcsf, Ccr2, Mcp1*, and *Tlr4*. Given the role of sex hormones in possible polarization and response, we also assessed the expression of sex hormone receptors—*Ar, Esr1*, and *Esr2—*in monocytes isolated from male and female mice. At 13 weeks of age, *Ar* expression was higher in males and *Esr2* expression was higher in females (**Figure 6A**). At 22 weeks of age, *Ar* and *Esr1* expression differed by sex, while *Esr2* expression was highest only in female monocytes stimulated with PA with a significant main effect of sex, diet, and interaction (**Figure 6B** and **Supplementary Table 3**). These results imply a role for age, sex, and sex receptors in monocyte phenotypic changes.

## Discussion

Investigating the mechanisms driving sexually dimorphic myeloid responses to HFD can help us in understanding the dimorphic physiology leading to differences in disease manifestation and treatment responses. In this study, we show that there are several pathways altered between male and female ATMs leading to differences in metabolism, inflammatory polarization, and function. Both ATM RNA-seq and monocyte migration studies demonstrate that while chemokine signals may differ by sex, monocytes themselves are also sexually dimorphic in their gene expression and function.

Our migration studies with monocytes are consistent with others showing male monocyte-derived macrophages to migrate readily to fatty acid exposure whereas female macrophages have dampened migration (39). While the literature is controversial about the importance of MCP1 in obesity-induced inflammation (47, 48), using the chemokine and PA treatment, we identified that male monocytes specifically have a more robust trafficking response *in vitro.* Sex differences in migration could be attributed to monocytes being heterogenous in nature with respect to the various chemokine and cytokine receptors where some are involved in tissue damage, while others promote tissue repair (49–51). MCSF facilitates tissue repair by recruiting monocytes to inflammation sites through CCR2 receptor activation and induction of CCL2/MCP1 production (52). Unlike the homeostatic role of MCSF, GMCSF shows pro-inflammatory effects by enhancing the survival and activation of recruited myeloid cells (53–55). CCR2 and CX3CR1 may synergistically impact the inflammatory monocyte phenotypes in obesity (56). It is highly likely that phenotypic changes in monocytes are sex-dependent with an increased burden on male monocytes for tissue recruitment. Interestingly, despite migratory responses, female monocytes failed to further upregulate the expression of inflammatory monocyte markers upon PA challenge (mimicking HFD), suggesting that female monocytes may be much more guarded and reparative in nature than males. In addition, the monocyte transfer studies (**Figure 4**) showed the inflammatory nature of male monocytes to produce CD11c^+^ ATMs even in female recipient mice. Also, we demonstrated the necessity of TLR4 in both male and female migratory responses similar to other studies showing that TLR4 signaling is critical for CCR2-dependent monocyte migration through binding of MCP1 to CCR2 in LPS studies (57), in angiogenesis and peripheral neuropathy (58). Our results emphasize that there are significant changes over the exposure of HFD and with age or even with loss of the TLR4 receptor that may alter chemokines and chemokine receptors consistent with the expression data in ATMs and that these changes are sexually dimorphic (**Figure 3**). Overall, these findings demonstrate that tissue recruitment signals as well as cellular receptors can play a crucial role in differentiating myeloid cells that recruit to metabolic tissues. This knowledge may play a critical role in identifying and treating individuals of both sexes at risk for metabolic inflammation.

In obesity, with ATM accumulation in the visceral depots, systemic inflammation is highly increased in males (59, 60) compared to females (16). While local proliferation of resident ATMs occur in both sexes (16), pro-inflammatory CD11c^+^ ATM numbers are higher in males than females even in old age (61), suggesting sex in addition to hormones influences macrophage phenotypes and metabolism. There are continued gaps in our understanding of why expansion of CD11c^+^ ATMs occurs primarily in male visceral tissue while subcutaneous depots are protected from this recruited inflammation. Our findings that fatty acids along with chemokines may drive monocyte recruitment might be an explanation for this crucial link between adipose lipid accumulation and the onset of meta-inflammation in humans and mouse (30, 62, 63). Our RNA-seq data demonstrated sex differences in key gene signatures of fat storage and inflammation pathways, implying a role for sex and sex hormones in regulating macrophage function. This is consistent with studies showing sex differences in lipid metabolism that regulate macrophage polarization (16, 17). A recent study using single-cell genomic analysis found that Trem2 signaling drives the formation of lipid-associated macrophages (LAMs) in crown-like structures in the visceral adipose tissue of males, preventing adipocyte hypertrophy and loss of systemic lipid homeostasis under obese conditions. The signature of Trem2 and associated genes involved in phagocytosis and lipid catabolism suggested that this pathway is a characteristic of a conserved macrophage response triggered by aberrations in lipid composition, levels, and distribution (64). Our data demonstrate a similar profile in males with HFD ATMs having increased *Lpl, Ctsb, and CD36* expression compared to female ATMs. Further studies are needed to determine sexual dimorphism in the development of these LAMs, given these changes in the metabolic profile (17). Also, the disparity in fat distribution (65) and the interplay between macrophage cellular metabolism and tissue niche during the development of obesity and its complications could be attributed to regulation of key proteins through sex steroid receptors present in adipocytes, ATMs, and circulating monocytes.

Limitations of our study are the focus on changes predominantly during HFD exposure and the focus on MCP1 in *in vitro* studies. Future studies are required to evaluate the role of other stimulatory conditions and chemokines, given the large range of ATM chemokines and receptors (**Figure 3**) that we have identified. Also, other leukocyte types vary by sex and contribute to these changes in meta-inflammation. For example, neutrophils from men also express higher levels of TLR4 and produce more TNF-α than female neutrophils both constitutively and following activation with LPS (66). Furthermore, while prior work has looked at the role of sex hormones in modifying responses (67), the role of sex hormones in the context of monocyte trafficking needs to be further studied. Consistent with a role for sex hormones, 17-β estradiol-treated macrophages have a reduced LPS response through controlling nuclear factor kappa-light-chain enhancer of activated B cell (NF-κB) intracellular localization (68). 17-beta estradiol has even been shown to exert an anti-inflammatory effect on adipocytes directly through reduced production of chemokines and NF-κB-mediated cytokines (69). In middle-aged male and female subjects, pro-inflammatory polarization of ATMs was observed by the presence of saturated fatty acids (SFAs) such as palmitate and palmitoleate, while alpha-linolenic acid, n-3 FAs, n-3/n-6 FA ratio, and eicosatetraenoic acid had the opposite effect in visceral WAT (70, 71). Other studies showed sex differences in lipid metabolism with the male GWAT showing increased accumulation of pro-inflammatory lipid metabolites such as ceramides, arachidonic acid, diacylglycerol, and phospholipids than the female GWAT (16, 22). Dietary interventions such as caloric restriction as well as dietary modifications can also alter monocyte inflammatory responses (72, 73). These findings imply a role for adipose lipid metabolism in modulating phenotypic changes in circulating and even BM monocytes that needs to be explored further.

Future studies will need to focus on determining both the role of sex on lipid composition and also on chemokine signaling and monocyte polarization as they differentiate into macrophages. Further studies with lipid mediators would be helpful to understand dietary patterns and inflammatory disease outcome in patients. Overall, investigating how sex hormone signaling affects monocyte development and monocyte heterogeneity will advance our understanding of sex differences in monocytic function at homeostasis and in the pathogenesis of diseases, which can ultimately impact future therapeutic design targeting monocytes in the clinic.

## Data Availability Statement

The datasets presented in this study can be found in online repositories. The names of the repository/repositories and accession number(s) can be found below: https://www.ncbi.nlm.nih.gov/, GSE181841.

## Ethics Statement

The animal study was reviewed and approved by the University of Michigan.

## Author Contributions

KS participated in hypothesis generation, experimental design, execution, data interpretation, and manuscript preparation and review. MV participated in experimental design, execution, data analysis, and manuscript preparation. MI performed RNA sequencing data analysis with R software. PS provided analytical statistical support. SA, JC, and AL participated in execution of experiments and manuscript preparation and review. All authors contributed to the article and approved the submitted version.

## Conflict of Interest

The authors declare that the research was conducted in the absence of any commercial or financial relationships that could be construed as a potential conflict of interest.

## Publisher’s Note

All claims expressed in this article are solely those of the authors and do not necessarily represent those of their affiliated organizations, or those of the publisher, the editors and the reviewers. Any product that may be evaluated in this article, or claim that may be made by its manufacturer, is not guaranteed or endorsed by the publisher.
