# Supplementary figures and images for: Monocyte Trafficking and Polarization Contribute to Sex Differences in Meta-Inflammation
Source: Front Endocrinol (Lausanne). 2022 Mar 28;13:826320. doi: 10.3389/fendo.2022.826320 (PMC9001155; doi:10.3389/fendo.2022.826320)

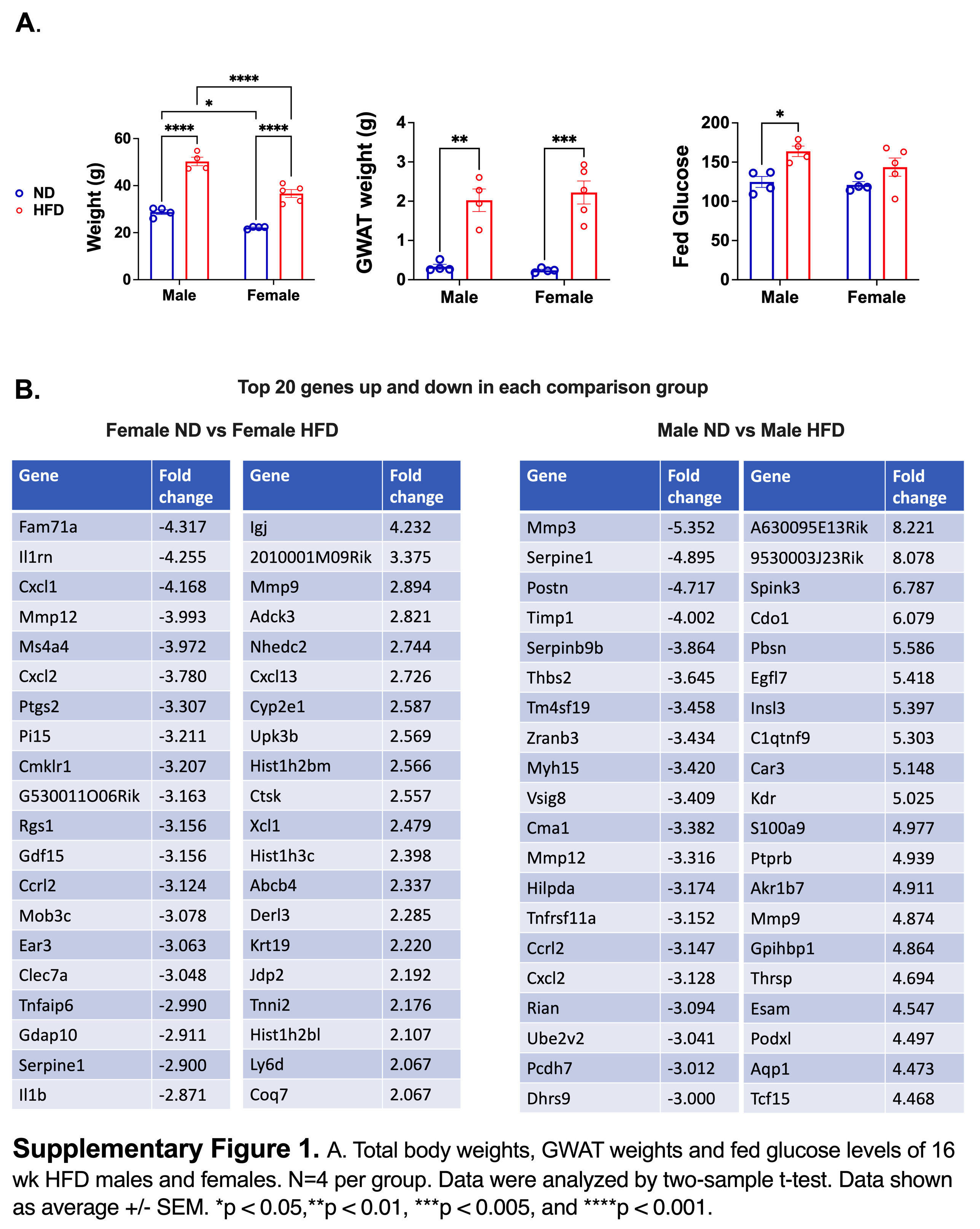

Supplement: Supplementary file 1 [file Image_1.tiff]

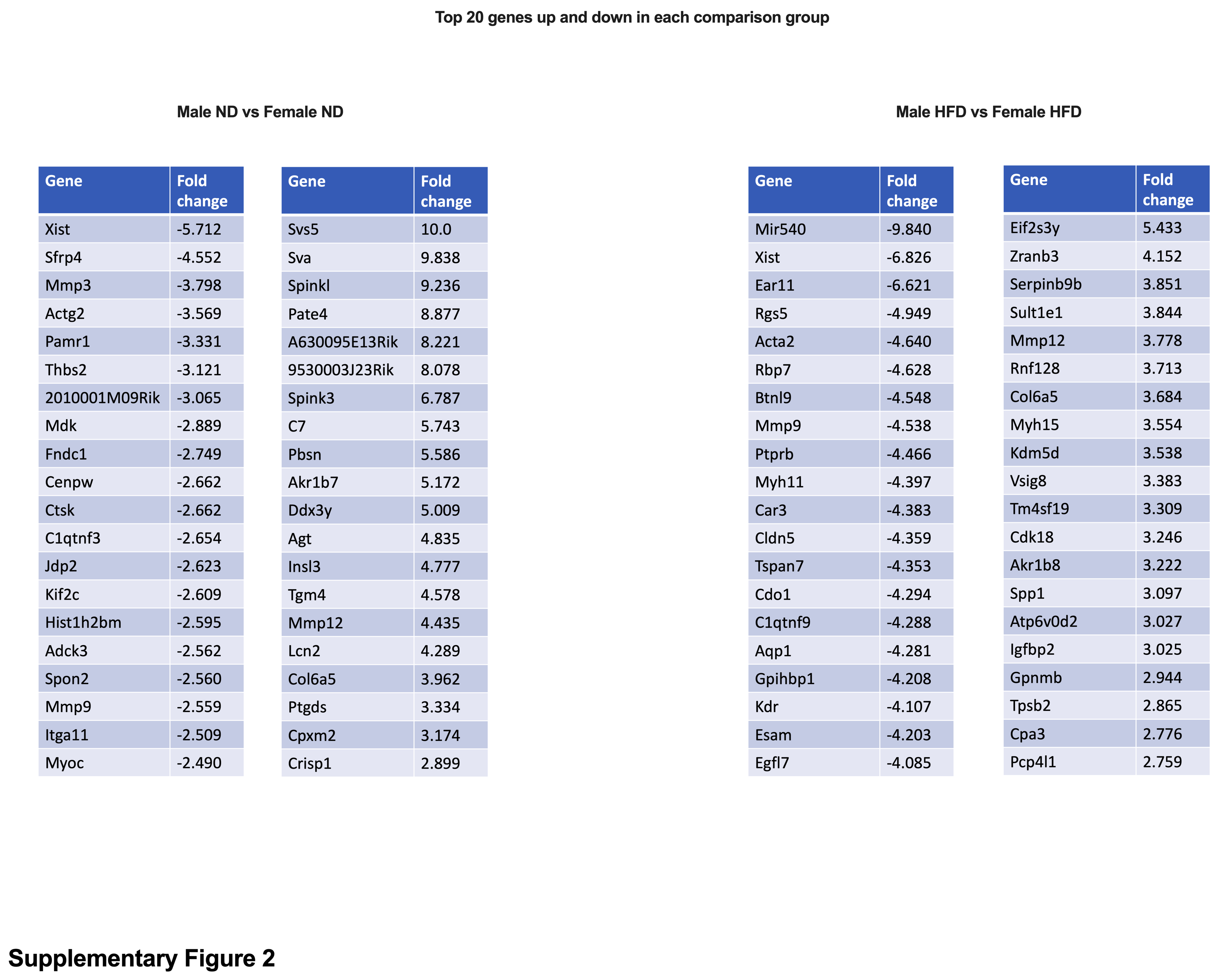

Supplement: Supplementary file 2 [file Image_2.tiff]
