# Supplementary material for: Monocyte Trafficking and Polarization Contribute to Sex Differences in Meta-Inflammation
Source: Front Endocrinol (Lausanne). 2022 Mar 28;13:826320. doi: 10.3389/fendo.2022.826320 (PMC9001155; doi:10.3389/fendo.2022.826320)
Supplement: Supplementary file 3 [file Table_1.docx]

|  | **Forward Primer (5’ to 3’)** | **Reverse Primer (5’ to 3’)** |
| --- | --- | --- |
| *Arbp* | AGATTCGGGATATGCTGTTGGC | TCGGGTCCTAGACCAGTGTTC |
| *Gmcsf* | ACCACCTATGCGGATTTCAT | TCATTACGCAGGCACAAAAG |
| *Mcsf* | GACTTCATGCCAGATTGCC | GGTGGCTTTAGGGTACAGG |
| *Tlr4* | ATGGCATGGCTTACCCACC | GAGGCCAATTTTGTCTCCACA |
| *Ccr2* | ATCCACGGCATACTATCAACATC | CAAGGCTCACCATCATCGTAG |
| *Cx3cr1* | GCAAGCTCACGACTGCCTTC | TCCGGTTGTTCATGGAGTTGG |
| *Mcp1* | TTAAAAACCTGGATCGGAACCAA | GCATTAGCTTCAGATTTACGGGT |
| *Ar* | TAGCAGGGCAGATCCTGTCT | GTAGACCCTTCCCAGCCCTA |
| *Esr1* | TGTGTCCAGCTACAAACCAATG | CATCATGCCCACTTCGTAACA |
| *Esr2* | CTGTGCCTCTTCTCACAAGGA | TGCTCCAAGGGTAGGATGGAC |

**Supplementary Table 1. Primer sequences**
