# Supplementary material for: Monocyte Trafficking and Polarization Contribute to Sex Differences in Meta-Inflammation
Source: Front Endocrinol (Lausanne). 2022 Mar 28;13:826320. doi: 10.3389/fendo.2022.826320 (PMC9001155; doi:10.3389/fendo.2022.826320)
Supplement: Supplementary file 4 [file Table_2.docx]

**Supplementary Table 2. Figure 3 statistical analysis**

| **Gene** | **Sex** | **Diet** | **Interaction** |
| --- | --- | --- | --- |
| *Cxcr2* | 0.421 | 0.968 | 0.119 |
| *Cxcr3* | 0.167 | 0.181 | 0.739 |
| *Cxcr4* | 0.268 | **0.009** | 0.342 |
| *Cxcr6* | 0.157 | 0.176 | 0.344 |
| *Cxcr7* | 0.000* | 0.055 | 0.124 |
| *Cx3cr1* | 0.019* | 0.156 | 0.178 |
|  |  |  |  |
| *Cxc11* | 0.216 | **0.000** | 0.831 |
| *Cxcl2* | **0.001** | **0.000** | **0.005** |
| *Cxcl9* | 0.348 | 0.562 | 0.076 |
| *Cxcl12* | **0.008** | **0.030** | 0.821 |
| *Cxcl13* | 0.498 | 0.172 | 0.665 |
| *Cxcl14* | 0.758 | **0.020** | 0.165 |
| *Cxcl16* | 0.115 | 0.123 | 0.284 |
|  |  |  |  |
| *Ccr1* | 0.274 | 0.864 | 0.661 |
| *Ccr2* | 0.654 | 0.058 | 0.732 |
| *Ccr3* | **0.011** | **0.022** | **0.028** |
| *Ccr4* | **0.044** | **0.049** | 0.893 |
| *Ccr5* | **0.001** | **0.001** | **0.041** |
| *Ccr6* | 0.074 | 0.620 | 0.372 |
| *Ccr7* | 0.514 | 0.357 | **0.031** |
| *Ccr8* | 0.057 | 0.170 | **0.003** |
| *Ccr9* | 0.132 | 0.250 | 0.868 |
| *Ccr10* | 0.513 | 0.460 | 0.172 |
| *Ccrl2* | **0.022** | **0.001** | 0.058 |
|  |  |  |  |
| *Ccl2* | 0.956 | **0.007** | **0.047** |
| *Ccl3* | **0.034** | **0.001** | 0.997 |
| *Ccl4* | 0.104 | **0.025** | 0.650 |
| *Ccl5* | 0.164 | 0.354 | **0.032** |
| *Ccl6* | 0.108 | **0.003** | 0.516 |
| *Ccl7* | 0.573 | **0.046** | 0.101 |
| *Ccl8* | 0.715 | **0.042** | 0.099 |
| *Ccl9* | **0.048** | **0.003** | 0.084 |
| *Ccl11* | 0.163 | **0.045** | 0.847 |
| *Ccl12* | 0.065 | **0.038** | **0.008** |
| *Ccl17* | 0.964 | 0.196 | **0.009** |
| *Ccl22* | 0.144 | 0.437 | **0.006** |
| *Ccl24* | 0.504 | 0.446 | **0.015** |
