# Supplementary material for: Monocyte Trafficking and Polarization Contribute to Sex Differences in Meta-Inflammation
Source: Front Endocrinol (Lausanne). 2022 Mar 28;13:826320. doi: 10.3389/fendo.2022.826320 (PMC9001155; doi:10.3389/fendo.2022.826320)
Supplement: Supplementary file 5 [file Table_3.docx]

**Supplementary Table 3. Figure 5 analysis**

|  | Sex  (F, M) | Treatment  (PA+MCP1) | Interaction |
| --- | --- | --- | --- |
| Lean | **0.000** | **0.000** | 0.056 |
| Lean TLR4^-/-^ | **0.000** | 0.096 | 0.585 |
| 2-4 week HFD | **0.020** | **0.000** | 0.300 |
| 6 week HFD | 0.235 | **0.000** | 0.158 |
| 12-16 week HFD | 0.057 | **0.000** | **0.012** |
