# Supplementary material for: Monocyte Trafficking and Polarization Contribute to Sex Differences in Meta-Inflammation
Source: Front Endocrinol (Lausanne). 2022 Mar 28;13:826320. doi: 10.3389/fendo.2022.826320 (PMC9001155; doi:10.3389/fendo.2022.826320)
Supplement: Supplementary file 6 [file Table_4.docx]

**Supplementary Table 4. Figure 6B analysis**

|  | Sex  (F, M) | Treatment  (PA+MCP1) | Interaction |
| --- | --- | --- | --- |
| *Mcsf* | **0.001** | 0.774 | 0.200 |
| *Gmcsf* | **0.000** | **0.014** | 0.056 |
| *Ccr2* | **0.023** | **0.026** | 0.940 |
| *Cx3cr1* | **0.000** | 0.151 | 0.702 |
| *Mcp1* | 0.354 | **0.005** | 0.329 |
| *Tlr4* | 0.584 | **0.028** | 0.642 |
| *Ar* | **0.041** | 0.185 | 0.073 |
| *Esr1* | **0.000** | 0.100 | 0.120 |
| *Esr2* | **0.000** | **0.000** | **0.029** |
